# How Physician—Insurance Contracting Contributes to the Medical Exodus and Access to Ophthalmic Care in Puerto Rico

**DOI:** 10.3390/epidemiologia5040050

**Published:** 2024-11-23

**Authors:** Luma Al-Attar, Rafael A. Ocasio Diaz, Andrea N. Ponce, Hossein Zare

**Affiliations:** 1 Retina Center of Puerto Rico, Manati, PR 00674, USA; rao4009@cornell.med.edu; 2 Department of Ophthalmology, Johns Hopkins Bloomberg School of Public Health, Baltimore, MD 21205, USA; 3 Department of Ophthalmology, University of Puerto Rico School of Medicine, Medical Sciences Campus, San Juan, PR 00921, USA; 4 Department of Population Health, Johns Hopkins Bloomberg School of Public Health, Baltimore, MD 21205, USA; aponce4@jhu.edu; 5 Department of Health Policy and Management, Johns Hopkins Bloomberg School of Public Health, Baltimore, MD 21202, USA; hzare1@jhu.edu

**Keywords:** insurance, contracting, Puerto Rico, Medicare Advantage

## Abstract

Background: Puerto Rico (PR) has experienced significant demographic changes, characterized primarily by an aging population and an unprecedented exodus of medical doctors. Ophthalmologists are of particular concern as they commonly serve older populations, and the island has high rates of some age-related eye diseases in the United States (US). Our research aims to investigate the factors driving ophthalmologists in PR to emigrate to the mainland US. Methods: This is a cross-sectional study among ophthalmologists in PR, using survey data collected from May to June 2023. This study recruited a convenient sample of all ophthalmologists practicing in PR via outreach in person and online communities. The survey covered various types of challenges faced by ophthalmologists, their demographics, and practice details. STATA/BE 18 statistical software was used for data analysis. Statistical tests, such as chi-square and proportion tests, were performed, stratifying results by age, gender, subspecialty, geographic health districts, experience, and practice type. Results: Among 130 of the estimated 218 ophthalmologists in PR, insurance/billing issues were identified as the primary challenge to practicing in PR and the primary reason to leave PR. The challenges that were identified included required authorizations for patient care, unjustified claim rejections, and threats of contract cancellation. We found that new ophthalmologists (≤15 years of practice) faced more specific challenges than experienced ophthalmologists (>15 years of practice), such as difficulty in obtaining insurance contracts. Conclusions: Insurance/billing issues are a pervasive concern for ophthalmologists in PR. New ophthalmologists are disproportionately affected by these challenges, potentially leading some to find employment outside of PR. There is a need for targeted policies—regulation of insurance contracting and increased reimbursement from private insurance plans—to reduce insurance contracting barriers for keeping a sustainable physician workforce in PR.

## 1. Introduction

Over the past 15 years, Puerto Rico (PR) has experienced two major demographic changes [1]. First, the population is getting older. From 2010 to 2021, the percentage of the elderly population (65+ years of age) on the island increased from 14.5% to 23% [2]. Second, many physicians are emigrating to the mainland United States (US) due to political, economic, and infrastructure challenges [3]. Since 2009, more than 9000 medical practitioners have left the island, resulting in a 46% reduction in physicians [4]. This has led to long wait times for physician appointments, difficulty finding specialist physicians, and reduced access to care for patients [5].

While practitioners of all specialties are affected by demographic shifts, ophthalmologists are of particular concern as most of their patients belong to the growing aging population. This could mean more age-related eye diseases, such as cataracts, macular degeneration, and diabetic retinopathy than the workforce can handle. PR already has one of the highest rates of visual impairment, diabetes, and diabetic retinopathy in the US [6,7,8]. Given the increasing demand for ophthalmic care and the decreasing supply of ophthalmologists, there is potential for a future reduction in access to care for older patients. This could lead to undiagnosed or untreated eye conditions, and thus higher prevalence of visual impairment [9].

The healthcare insurance infrastructure in PR may be a contributing factor to the medical exodus. Approximately 95% of PR residents have some form of health insurance including Medicare (with 94% Medicare Advantage penetration) [10], local commercial plans (Triple S, Medical Card System, First Medical), and local indigent care partially funded by Medicaid (Plan Vital). However, in PR, reimbursement for physicians is generally lower compared to the mainland US despite the increased cost of importing medical supplies and the burden of an 11.5% sales tax [11]. It is important to note that while Medicare is typically the lowest payor in the mainland US [12], it is the highest payor in PR [13]. Moreover, commercial plans in PR reimburse physicians at an average rate of USD 26 per visit [11], while in the mainland US rates range from USD 76 to USD 199 [14]. Medicare Advantage plans in PR, which include Management Service Organization of PR (MSO) (Medicare y Mucho Más (MMM)/Premier Medicare Choice (PMC)), Medical Card System (MCS) Classicare, and Seguros de Servicios de Salud (SSS or Triple S) Advantage, often reimbursed for below Medicare rates or offer capitation contracts [5], further exacerbating the reimbursement challenges faced by physicians. Fear of legal retaliation from insurance plans and government agencies, such as the Federal Trade Commission (FTC), provides another hurdle for physicians in PR. Physicians are hesitant to speak out against coercive policies made by Medicare Advantage and other commercial insurance plans [15]. Physicians are subject to anti-trust laws and have previously been apprehensive that any communication with their peers about mutual insurance and billing concerns could be wrongly perceived as “collective bargaining”, thus violating anti-trust laws [16]. In 2017, a Medicare Advantage plan filed a complaint against the ophthalmology cooperative in PR for collective bargaining [17]. Limiting the important flow of information among physicians is especially damaging to ophthalmologists in PR who are mostly solo practitioners and further isolates individual ophthalmologists facing powerful insurance plans. Meanwhile, insurance companies are one of the few industries exempt from anti-trust laws in the US according to the 1945 McCarran-Ferguson Act. In PR, this means that the four commercial plans dominating the market—MSO (MMM and PMC Advantage), MCS, Triple S, and First Medical—can share information and strategies [18].

The motives behind the medical exodus of specialists leaving PR are multifactorial and related to the history and political status of PR, such as high poverty rates, poor infrastructure, and vulnerability to natural disasters. After Hurricane Maria in 2017, there was an increased exodus of PR residents [19] and damage to the healthcare infrastructure [20]. It also exposed the vulnerability of the healthcare system given the medical exodus and led the PR government to institute the tax incentive for specialty physicians in an attempt to retain them [21]. Varas-Diaz et al. conducted a qualitative study of 50 physicians who had left PR or decided to stay in PR to understand the role of coloniality in this exodus and found three themes of concern: “the combination of the overall social deterioration of the Puerto Rican setting, a healthcare system impacted by political corruption, and a bleak outlook for medical students and new professionals with shrinking opportunities and resources on the Island” [22]. However, little data are available from physicians who have chosen to stay in PR.

To date, the only policy to retain specialists has been an income tax incentive (Acts No. 14 in 2017 (introduction), No. 60 in 2019 (codification), and Act No. 47 in 2020 (expansion) [21]. One important factor that has not been fully explored is the role of insurance contracting, insurance plan policies, and low reimbursement in potentially driving away new physicians entering the workforce in PR. In 2012, a survey commissioned by the Colegio de Médicos Cirujanos de PR found that 90% of physicians in the study who were considering leaving PR reported lower reimbursement rates to be one of the main factors behind leaving the island [11]. To further explore this and other concerns, this current research aims to describe the challenges that ophthalmologists are facing in PR including insurance/billing, economic, patient care, infrastructure, natural disaster, and personal challenges. Insights regarding the challenges that ophthalmologists face will provide guidance on the resources and support they need to meet current and future healthcare demands.

## 2. Materials and Methods

### 2.1. Overview

An island-wide cross-sectional study was conducted among ophthalmologists in PR. The inclusion criteria included all ophthalmologists actively practicing in PR and the exclusion criteria included non-ophthalmologists, any ophthalmologist, not actively practicing in PR, and ophthalmologists in PR who were still in residency or fellowship training programs.

Using the Cochran formula with α = 0.05 (95% CI and β = 0.8 (power), and considering 41% of the physicians have left the workforce in PR [23], we computed 147 as the study sample size. We distributed the survey to 218 ophthalmologists in PR, and we received 130 completed surveys, surveys were collected from May to June 2023. This study recruited a convenient sample of all ophthalmologists practicing in PR via outreach in person and online communities. First, an author (ROD) presented at the Sociedad Puertorriqueña de Oftalmología (SPO) Annual Convention where he introduced the study and invited ophthalmologists to participate. Second, an author (LA) recruited participants via the SPO email listserv. Third, we used various ophthalmology WhatsApp chats (e.g., Eye Care Puerto Rico and Women in Ophthalmology), a popular instant messaging application among many ophthalmologists in PR, to reach those who did not attend the SPO Annual Convention or were not SPO members. A link to the study survey was provided in the email and text message exchanges. Based on the National Provider Identifier Database, there were 218 ophthalmologists practicing in PR as of 25 May 2023 [24].

### 2.2. Survey

Two authors (LA, ROD) designed a survey based on their personal experiences and commonly discussed topics and issues among ophthalmologists practicing in PR. A pilot survey was sent out to six ophthalmologists of varying specialties, ages, and geographic health districts for feedback. Their comments were incorporated into the final survey, and they later completed the survey as study participants. Using data from six pilot participants, the reliability of questions was assessed and yielded a Cronbach’s alpha coefficient of 0.78. The anonymous nature of the survey was important as it allowed respondents to freely express their concerns without fear of retaliation or criticism. The survey consisted of 27 close-ended questions about demographics (e.g., age group, years of practice, gender), practice (e.g., geographic health districts, subspecialty, type), patient volume, and challenges divided by category (e.g., economic, insurance/billing, patient care, infrastructure, natural disaster, personal). For each category, participants were asked to select from a broad list of challenges that applied to them. Examples of billing/insurance plan challenges included the need for referral/authorization for patient care, delay in payment from insurance companies, and annual reduction of reimbursement for services from Medicare Advantage and other payors. Participants were prompted to rank the six types of challenges, from most to least important, in terms of challenges to practicing in PR and then in terms of challenges that would lead them to leave PR. The last section of the survey covered questions related to leaving PR. For example, we asked participants whether they had seriously considered leaving PR and practicing elsewhere or retiring since 2017 and to rank solutions that the PR government could do to best retain ophthalmologists in PR. At the end of the survey was an open-ended question for participants to submit questions, comments, and recommendations.

### 2.3. Statistical Analysis

A descriptive analysis was conducted to generate frequencies for demographic variables and insurance-/billing-related measures. In this study, we defined “new” ophthalmologists as having 15 years or less of experience and “experienced” ophthalmologists as having more than 15 years of experience. Previous studies have found that 52% of ophthalmologists in the US in 2003 planned to retire after the age of 65 [25], and 544% in 2021 were at least 55 years old [26]. Additionally, based on common knowledge among the ophthalmology community, most enter the workforce at the age of 30–35. This observation and the aforementioned findings from the literature suggest that the average ophthalmology career lasts at least 30–35 years, providing the basis for the 15-year cutoff. Differences between new and experienced ophthalmologists in insurance/billing measures were examined using proportion tests for binary variables, chi-square tests for categorical variables, and Fisher’s exact tests for categorical variables with less than 5 in a cell. All analyses were carried out using STATA/BE Version 18 (StataCorp, LLC, College Station, TX, USA). The significance level was considered at *p* < 0.05 and one-tailed.

The study was approved by the Institutional Review Board (blinded for review purposes). The researchers adhered to the tenets of the Declaration of Helsinki.

## 3. Results

### 3.1. Characteristics of Participants

The survey had a response rate of 60%, representing 130 of the estimated 218 ophthalmologists on the island during data collection [24]. The sample represented different age groups, genders, geographic health districts, subspecialties, and types of practices overall and was stratified by years of practice (new: n = 47; experienced: n = 83) (Table 1). A majority of participants were over 44 years of age (71%), were male (64%), had practices in the Metro Area (54%), practiced as comprehensive ophthalmologists (general) (35%), and had solo practices (50%). Lastly, 33% of respondents reported considering leaving PR or retiring since 2017.

### 3.2. Insurance/Billing Challenges

Table 2 presents insurance/billing measures that were assessed and stratified by years of practice. Insurance/billing was the most common number-one challenge to practice in PR (58%) and the number-one reason to leave PR (37%). Insurance/billing challenges were ranked number one among all age groups, for both genders, all geographic health districts, most subspecialties, and those in private (solo or group) practice. A significantly higher proportion of new ophthalmologists ranked insurance/billing challenges as the number one challenge to practicing in PR and the number one reason that would lead them to leave PR compared to experienced ophthalmologists (*p* < 0.05).

Commonly reported insurance/billing challenges included the need for referral/authorization for necessary treatments (74%), unjust claims rejections (71%), and lack of or difficulty in obtaining insurance approval for new treatments (69%) (Table 2). There were concerns about the annual reduction in reimbursement rates, both at the federal level (Medicare) (61%) and even more so locally among Medicare Advantage plans (66%).

The top three best payors were reported as MCS Classicare Advantage, Medicare (First Coast), and Triple S Advantage, while the top three worst payors were First Medical Commercial, Triple S Commercial, and MMM Advantage. Significantly more men reported MCS Classicare Advantage as their best payor compared to women (*p* < 0.05) who most commonly reported Medicare (First Coast) as their best payor.

### 3.3. Insurance–Physician Contracts

Several common concerns were related to insurance–physician contracts such as feeling that the power of insurance companies forced them into a contract lower than Medicare fees (65%) and feeling threatened that the insurance plans could cancel their contract (35%) (Table 2). When asked specifically about insurance–physician contracts (Table 3), many reported experiencing unjust cancellation of their contract (25%) and difficulty obtaining a contract (64%). Significantly more new ophthalmologists faced difficulty getting a health insurance contract than experienced ophthalmologists (*p* < 0.05). Ophthalmologists who had considered leaving PR were more likely to have their insurance contract canceled than those that had not considered leaving PR (*p* < 0.05).

Insurance plans that posed barriers to contracting varied based on the ophthalmologist’s years of practice (Table 3). New ophthalmologists were significantly more likely to report difficulty getting a contract with a Medicare Advantage plan compared to experienced ophthalmologists (*p* < 0.05). Of the three most common Medicare Advantage plans in PR (MSO, which includes MMM and PMC, Triple S Advantage, and MCS Classicare Advantage), MSO posed the biggest barrier to physician contracting overall and particularly among new ophthalmologists (39%).

A majority of participants reported having contracts with Medicare Advantage (82%), Medicare (First Coast) (81%), and commercial insurance plans (81%) (Table 3). Significantly more new ophthalmologists than experienced ophthalmologists (*p* < 0.05) had a contract with Plan Vital.

### 3.4. Medicare Advantage Volume

When asked about how Medicare Advantage or Plan Vital affected their patient volume, there were differences among new and experienced ophthalmologists. About 76% of survey participants reported that more than 50% of their patient volume consisted of Medicare Advantage enrollees, of which 44% reported more than 75% of their patient volume consisted of Medicare Advantage enrollees. Significantly more experienced ophthalmologists reported that Medicare Advantage represented over 75% of their practice volume than their less experienced counterparts (*p* < 0.05). Meanwhile, experienced ophthalmologists were more likely than new ophthalmologists to report that Plan Vital represented less than 10% of their volume.

### 3.5. Policy Solutions

Among 105 participants, income tax incentives were ranked as the number one government solution to best retain ophthalmologists in PR (39%), followed by regulating insurance company contracting to protect physicians (38%) (Table 3). New ophthalmologists were significantly more likely than experienced ophthalmologists (*p* < 0.05) to rank increasing reimbursement from private insurance plans as their number one policy solution.

### 3.6. Utilization of Income Tax Incentive

Survey respondents reported on their current participation in the government’s income tax incentive to retain specialty physicians in PR. About 71% of the sample was utilizing the government income tax incentive at the time of the survey, of which 78% found it to be “somewhat or very important” in their decision to continue practicing in PR. Ophthalmologists aged 25–44 were significantly more likely than ophthalmologists aged 45 or older (*p* < 0.05) to report that the tax incentive was “very important” in their decision to continue practicing in PR.

## 4. Discussion

The present study is the first research to explore the challenges ophthalmologists in PR are facing and the reasons that would lead them to leave PR. Among 130 ophthalmologists who responded to the study survey between May to June 2023, we found that economic and insurance/billing factors were the major issues for practicing in and leaving PR, especially for new ophthalmologists. Insurance contracting, especially among Medicare Advantage plans, and unjust cancellations of insurance–physician contracts were common barriers, with new ophthalmologists experiencing these barriers more often than those who were experienced. Medicare Advantage plans were found to have an important role in patient volume for ophthalmologists, shedding light on the potential influence of such plans on the practice of ophthalmology in PR.

Given the 94% Medicare Advantage penetration in PR [10], many physicians are left with limited reimbursement options and thus settle for contracts with reduced reimbursement rates [5]. The lower reimbursement for physicians in PR compared to the mainland US is multifactorial. Medicare Advantage plans in PR receive 38% less compensation per beneficiary from the federal government than the mainland US [27]. Local private insurers are also collecting lower premiums than the mainland US [28], which in turn leads to lower provider fees. Finally, the two funding sources for Plan Vital, the government-funded insurance for the indigent, are limited. The federal government’s contributions are based on the Federal Medical Assistance Percentage (FMAP) and unlike other states, it is currently capped at 76%, resulting in less relative federal funding than the mainland US [29]. The second source of funding is the local government. However, their resources are limited as they recently faced bankruptcy, restricting available funding to pay health providers [30]. These disparities in reimbursement rates and the challenges faced by physicians highlight the need for policy changes and interventions to address the inequities in reimbursement between PR and the mainland US.

Despite the economic, infrastructure, patient care, personal and natural disaster factors—exacerbated by Hurricane Maria—that have affected life and ophthalmic practice in PR, we identified the elephant in the room: the intimidating health insurance plans that set roadblocks to physician contracting and reimbursement. In the present study, insurance/billing challenges were important to practicing and staying in PR and were commonly ranked as the number one challenge and the number one reason to leave PR overall in the sample and when stratified by subgroups. These findings support results from a 2012 survey by the Colegio de Médicos Cirujanos de PR identifying reimbursement as an important factor for physicians considering emigration [11]. Frustration with insurance plans and billing noted in this study was consistent with surveys of physicians and ophthalmologists in the US, such as a 2008 study by the Physicians Foundation, which found that reimbursement was ranked highest among physicians in what they liked least about medicine [31]. Additionally, according to a report in January 2023 by Ophthalmology Breaking News, a LinkedIn survey of ophthalmologists found that the top challenge they faced was reimbursement challenges which was reported at 40% [32]. Other studies have reported insurance and billing challenges as an important source of burnout [33,34,35] and stress [36] among physicians in the US.

To reduce the medical exodus and maintain a sufficient ophthalmology workforce, attention needs to be drawn to challenges faced by new ophthalmologists, especially the insurance/billing issues we found as the number one challenge and number one reason to leave PR. It is important to attract and retain new ophthalmologists who will serve the community longer than more experienced physicians who are closer to retirement [3]. Our findings found that new ophthalmologists disproportionately face particular challenges such as barriers to insurance contracting. This finding is consistent with a study among physicians who left or remained in PR, which shows the need for the government to better police insurance companies and ensure timely contracting of new providers [37]. These contracting hurdles and delays may pose a threat to the ophthalmology workforce as difficulty in obtaining an insurance–physician contract could hinder new ophthalmologists from choosing to practice in PR.

In PR, Medicare Advantage plans play a pivotal role in the ophthalmology workforce as 94% of Medicare beneficiaries have Medicare Advantage [10]. Both the PR College of Physicians and Surgeons, as well as investigative reporters, have described anecdotal evidence of the Medicare Advantage plans’ manipulative contracting techniques leading to fewer contracted physicians and lower reimbursement [5,38]. The present study indicates that almost half of new ophthalmologists in PR had difficulty getting a contract with a Medicare Advantage plan, particularly with MSO (MMM/PMC; 38%) and Triple S Advantage (29%). Furthermore, 38% of surveyed ophthalmologists said that the most important policy to retain ophthalmologists in PR would be to regulate insurance company contracting to protect physicians.

Barriers to insurance contracting might not only deter new ophthalmologists from practicing in PR but might also reduce patient access to care. In this study, 10% of new ophthalmologists also reported difficulty obtaining a contract with commercial plans (significantly more than experienced physicians) and 6% reported difficulty getting a contract with Plan Vital to serve the indigent population. This is especially important as new ophthalmologists play an important role in serving indigent patients with Plan Vital plans. We found that new ophthalmologists were more likely to have a contract with Plan Vital and that more of their practice volume consisted of Plan Vital. To address the barriers new doctors face in obtaining insurance–physician contracts, legislation was passed in August 2023 to expedite and centralize credentialization for new physicians in PR for insurance-provider contracts, which can currently take up to one year [39]. Although the legislation was intended to expedite the credentialing process for a physician to obtain an insurance contract, it does not increase the number of physician contracts offered, increase reimbursement rates, reduce capitation, regulate payments, or prevent unjustified contract cancellations. While it is a step in the right direction, it is too soon to determine the effects of this legislation.

The present study provides insights into the influence of Medicare Advantage plans in the ophthalmology market in PR, given the 94% penetration of Medicare beneficiaries [10]. There are predominantly three Medicare Advantage plans: MSO (MMM/PMC), MCS Classicare, and Triple S Advantage. In this sample, 58% of ophthalmologists reported that over half of their patient volume came from Medicare Advantage plans; 34% reported that Medicare Advantage plans comprised over 75% of their patient volume. Given anti-trust laws and the prevalence of solo practitioners in PR, physicians are unable to voice and discuss insurance contracting concerns officially or unofficially. Furthermore, they are afraid of repercussions from insurance companies who could cancel their contracts. About 20% of ophthalmologists in the present study reported unjust cancellations of their insurance contract (and these ophthalmologists were more likely to report considering leaving PR compared to those who did not report unjust cancellation) and 35% reported feeling a threat of insurance contract cancellation. Given that there are only three main Medicare Advantage plans, ophthalmologists risk losing up to 30% of their income if they voice concerns about Medicare Advantage insurance contracts or rates. Also, the much higher prevalence of solo practitioners in PR gives them less negotiating power when handling insurance–physician contracts or reimbursement issues. These factors may explain the observation that ophthalmologists who had experienced an unjust contract cancellation were more likely to report considering leaving PR.

Our findings provide evidence to support a growing body of literature indicating that Medicare Advantage plans may reduce physician reimbursement [40] as two of the three Medicare Advantage plans were less commonly reported than Medicare to be the best payor by survey respondents. Furthermore, one Medicare Advantage plan, MSO, was ranked as the third most common worst payor overall. This finding supports Herndon’s theory that health insurance monopsony (only one buyer) can lead to lower physician reimbursement rates [16]. Low reimbursement rates among all payors were reported as a challenge by ophthalmologists in this survey, which is consistent with concerns reported by ophthalmologists across the US [32]. However, ophthalmologists in PR may be even further affected for several reasons. First, the federal Medicare fee schedule in PR is less than in the mainland US, potentially driving them to leave PR. Second, Medicare Advantage plans in PR have the highest penetration in the US at 94% [10], and in this survey, commercial payors were reported by ophthalmologists to be the most common worst payors.

Interestingly, one gender discrepancy was noted when reporting the best payor. In the present survey, male ophthalmologists were more likely than female ophthalmologists to report a Medicare Advantage plan (MCS Classicare) to be their best payor, suggesting that men were more likely than female ophthalmologists to negotiate a contract with this Medicare Advantage plan at rates above the Medicare fee schedule than the women ophthalmologists. This finding should be explored in further research but is consistent with evidence of gender pay gaps among physicians and ophthalmologists in the US [41].

Ophthalmologists in the present study voiced concern about limitations to patients’ access to care by insurance plans as 74% of ophthalmologists reported frustration with the need for referrals/authorization for necessary treatments. Additionally, about 69% of study participants had difficulty getting approval for new treatments, making it more difficult for ophthalmologists in PR to offer the standard of care, thus compromising the care of patients in their communities. This is consistent with similar findings among physicians in the US as the Physicians Foundation found that 76% of physicians had reported that insurance requirements reduced their ability to provide high-quality and cost-efficient care [42].

In an effort to retain specialists and increase care for the indigent population, the PR government passed an income tax incentive [21]. This survey provides important and novel information about the utilization of the tax incentive for ophthalmologists in PR. There was high utilization of the tax incentive among ophthalmologists (71%) and more than a majority reported the policy to be very important in retaining specialists. Younger ophthalmologists (aged 25–44) were more likely than older ophthalmologists (aged 45 or above) to report that the tax incentive as “very important”. While tax incentives work, they may not be enough.

This research has several limitations. First, the cross-sectional nature of the data that were collected does not allow us to establish temporality. Second, while the findings of this study could provide insight into other medical specialties in PR and the medical exodus in general in PR, it is limited to ophthalmologists and is further limited by the selection bias of those ophthalmologists who chose to participate in the survey. Thus, the generalizability of study findings may not extend to other medical specialties. Furthermore, this study only includes the perspective of physicians who have chosen to stay in PR. Without a group of ophthalmologists who chose to leave PR, we could not compare the challenges they face. Future research efforts could collect data from ophthalmologists and other physicians who have left PR to gain a deeper understanding of the medical exodus. Qualitative interviews with ophthalmologists who stayed in PR could also identify factors that influenced their decision to stay to potentially attract more physicians to PR.

Future policies need data to identify the root causes of the problem and to measure the efficacy of their implementation. This survey is the first known survey to ask specialists in PR what health policies they would recommend in order to retain them in PR. The most common number one policy recommendations were income tax incentives, regulation of insurance company contracting to protect physicians, and increasing reimbursement from private insurance including Medicare Advantage.

Despite current legislation to facilitate and encourage the availability of new insurance contracts, insurance contracting remains a barrier for new ophthalmologists in PR, potentially reducing patient access to care. Policymakers may consider modifying the legislation and or monitoring its execution to address the challenges that new ophthalmologists are facing in PR.

## 5. Conclusions

Insurance/billing issues were a pervasive concern faced by ophthalmologists in PR. New ophthalmologists were disproportionately affected by these challenges, potentially leading some to find employment opportunities outside of PR. There is a need for targeted policies-regulation of insurance company contracting, and increased reimbursement from private insurance plans to reduce insurance contracting barriers for keeping a sustainable physician workforce in PR.

## Figures and Tables

**Table 1:** Characteristics of the sample (n = 130).

	All	Years in Practice ^c^		
		New	Experienced	
	N (%)	n (%)	n (%)	*p*-Value
Age				<0.001 *^,d^
25–44	38 (29)	38 (81)	0 (0)	
45+	92 (71)	9 (19)	83 (100)	
Gender ^a^				0.167 ^e^
Male	83 (64)	26 (57)	57 (69)	
Female	46 (36)	20 (43)	26 (31)	
Health district ^b^				
Metro Area	70 (54)	22 (47)	48 (58)	0.226 ^e^
District of Arecibo	22 (17)	11 (23)	11 (13)	0.138 ^e^
District of Caguas	17 (13)	6 (13)	11 (13)	0.937 ^e^
District of Mayagüez	15 (12)	6 (13)	9 (11)	0.742 ^e^
District of Bayamón	13 (10)	-	-	-
District of Ponce	10 (8)	-	-	-
District of Fajardo	11 (8)	-	-	-
Subspecialty ^b^				
Comprehensive (General)	45 (35)	6 (13)	39 (47)	<0.001 *^,e^
Retina	31 (24)	19 (40)	12 (14)	0.001 *^,e^
Glaucoma	19 (15)	11 (23)	8 (10)	0.033 *^,e^
Cornea/Anterior Segment	15 (12)	-	-	-
Refractive	11 (8)	-	-	-
Pediatric	5 (4)	-	-	-
Neuro-ophthalmology	5 (4)	-	-	-
Uveitis	5 (4)	-	-	-
Oculoplastics	5 (4)	-	-	-
Type of Practice ^b^				
Solo practitioner	65 (50)	12 (26)	53 (64)	<0.001 *^,e^
Small group (<5 ophthalmologists)	48 (37)	27 (57)	21 (25)	<0.001 *^,e^
Large group (5+ ophthalmologists)	18 (14)	10 (21)	8 (10)	0.065 ^e^
University	14 (11)	-	-	-
Veterans Affairs Hospital	7 (5)	-	-	-

^a^ Excludes missing (n = 1); ^b^ May select more than one; ^c^ Column percentages; ^d^ Fischer’s exact tests; ^e^ Chi-square tests; Suppressed data that contained less than 5 participants in at least one study group (marked as -); * *p* < 0.05.

**Table 2:** Insurance/billing challenges among ophthalmologists in Puerto Rico (PR).

	All	Years in Practice ^b^		
		New	Experienced	
	N (%)	n (%)	n (%)	*p*-Value ^c^
Insurance/billing ranked as #1 challenge	61 (58)	28 (76)	33 (48)	0.003 *
Insurance/billing ranked as #1 reason to leave PR	37 (37)	21 (62)	16 (25)	0.0001 *
Insurance/billing challenges ^a^				
Need for referral/authorization for patient care	96 (74)	35 (74)	61 (73)	0.452
Unjustified rejection	92 (71)	31 (66)	61 (73)	0.818
Lack of or difficulty in obtaining insurance approval for new treatments	90 (69)	32 (68)	58 (70)	0.584
Annual reduction of reimbursement for services from Medicare Advantage (specific to PR)	86 (66)	26 (55)	60 (72)	0.025 *
Power of insurance companies forcing you into a contract that is less than Medicare fees	85 (65)	30 (64)	55 (66)	0.610
Delay in payment from insurance companies	82 (63)	30 (64)	52 (63)	0.447
Annual reduction of reimbursement rates for services from federal Medicare (First Coast)	79 (61)	27 (57)	52 (63)	0.720
Difficulty communicating with personnel at insurance companies for any rejections	71 (55)	22 (47)	49 (59)	0.911
Annual reduction of fees from commercial insurance companies in PR	64 (49)	25 (53)	39 (47)	0.248
Threat of cancellation of insurance contract	45 (35)	14 (30)	31 (37)	0.808
Annual reduction of fees from Plan Vital	35 (27)	17 (36)	18 (22)	0.037 *

^a^ May select more than one; ^b^ Column percentages; ^c^ Proportion tests; * *p* < 0.05.

**Table 3:** Insurance/billing contracting among ophthalmologists in Puerto Rico (PR).

	All	Years in Practice ^b^		
		New	Experienced	
	N (%)	n (%)	n (%)	*p*-Value
Has a contract with ^a^				
Medicare Advantage	107 (82)	36 (77)	71 (86)	0.901 ^c^
Medicare (First Coast)	105 (81)	36 (77)	69 (83)	0.818 ^c^
Commercial	105 (81)	36 (77)	69 (83)	0.818 ^c^
Plan Vital	81 (62)	35 (74)	46 (55)	0.016 *^,c^
Patient Volume: Plan Vital				0.005 *^,d^
0–10%	31 (32)	6 (17)	25 (40)	
11–25%	39 (40)	13 (37)	26 (42)	
26–50%	24 (25)	13 (37)	11 (18)	
51–75%	3 (3)	3 (9)	0 (0)	
76–100%	0 (0)	0 (0)	0 (0)	
Patient Volume: Medicare Advantage				0.014 *^,d^
0–10%	4 (4)	2 (6)	2 (3)	
11–25%	3 (3)	0 (0)	3 (4)	
26–50%	18 (18)	9 (26)	9 (13)	
51–75%	32 (32)	15 (44)	17 (25)	
76–100%	44 (44)	8 (24)	36 (54)	
Ever had difficulty getting a health insurance contract	69 (64)	36 (95)	33 (47)	<0.001 *^,c^
Had difficulty getting contract with ^a^				
Medicare Advantage	25 (29)	15 (48)	10 (18)	0.002 *^,c^
MSO (MMM and PMC Advantage)	19 (22)	12 (39)	7 (13)	0.003 *^,c^
Triple S Advantage	11 (13)	9 (29)	2 (4)	<0.001 *^,c^
MCS Classicare Advantage	5 (6)	3 (10)	2 (4)	0.125 ^c^
Commercial	3 (3)	3 (10)	0 (0)	0.009 *^,c^
Plan Vital	5 (6)	2 (6)	3 (5)	0.425 ^c^
Ever had health insurance contract wrongly cancelled	27 (25)	8 (21)	19 (26)	0.732 ^c^
Ranked #1 policies to retain ophthalmologists in PR				
Income tax incentives	41 (39)	9 (24)	32 (47)	0.011 *
Regulate insurance contracting to protect physicians	40 (38)	14 (38)	26 (38)	0.516
Increase reimbursement for private insurance plans	23 (22)	13 (35)	10 (15)	0.008 *

^a^ May select more than one; ^b^ Column percentages; ^c^ Proportion tests; ^d^ Fischer’s exact tests; * *p* < 0.05.

## Data Availability

The data presented in this study are available on request from the corresponding author due to privacy reasons.
